# Pneumocystis pneumonia induced by treatment with low-dose tacrolimus and methylprednisolone in a patient with rheumatoid arthritis: a case report

**DOI:** 10.1186/1756-0500-6-498

**Published:** 2013-12-01

**Authors:** Motoyasu Kato, Kazunori Tobino, Yuichi Fujimoto, Isao Kobayashi, Koji Sugano, Hitoshi Tokuda, Hiroki Ienaga, Kazuhisa Takahashi

**Affiliations:** 1 Department of Respiratory Medicine, Juntendo University Graduate School of Medicine, 2-1-1 Hongo, Bunkyo-ku, Tokyo 113-8421, Japan; 2 Department of Respiratory Medicine, Koshigaya Municipal Hospital, Koshigaya city, Saitama, Japan; 3 Department of Respiratory Medicine, Social Insurance General Central Hospital, Tokyo, Japan

**Keywords:** Pneumocystis pneumonia, Tacrolimus, Rheumatoid arthritis

## Abstract

**Background:**

Tacrolimus is an immunosuppressive drug used to prevent acute rejection following organ transplantation and to treat autoimmune disease. Tacrolimus is usually prescribed in such situation at a dose of 3.0 mg/day. Pneumocystis pneumonia induced by this dose of tacrolimus has been reported in many cases; however, we encountered a rare case of Pneumocystis pneumonia induced by low-dose tacrolimus and methylprednisolone.

**Case presentation:**

We herein report the case of an 82-year-old Asian Japanese female with rheumatoid arthritis and Pneumocystis pneumonia who was being treated with low-dose tacrolimus and low-dose methylprednisolone therapy. She was diagnosed with rheumatoid arthritis at 52 years of age and was administered oral low-dose methylprednisolone and salazosulfapyridine. Her condition had been stable under this treatment for 30 years. However, her arthralgia worsened three months before admission. The salazosulfapyridine was changed to tacrolimus (0.5 mg/day) by her physician, and her arthralgia almost completely disappeared. She was admitted to our hospital for Pseudomonas pneumonia, and her symptoms improved almost completely with intravenous ceftazidime therapy. However, on the 14th day of admission, she developed acute respiratory failure due to Pneumocystis pneumonia and died on the 17th day of admission in spite of adequate treatment.

**Conclusion:**

Our report highlights the importance of providing prompt prevention, diagnosis and treatment of Pneumocystis pneumonia in rheumatoid arthritis patients under tacrolimus and low-dose methylprednisolone therapy.

## Background

Tacrolimus is an immunosuppressive drug often administered to prevent acute rejection following organ transplantation and to treat autoimmune diseases (e.g., rheumatoid arthritis (RA), polymyositis/dermatomyositis and ulcerative colitis (UC)) and atopic dermatitis. Tacrolimus is usually administered at a dose of 3.0 mg/day for these diseases. This dose of tacrolimus can potentially generate an immunocompromised status, leading to opportunistic infections (e.g., fungal infection, Pneumocystis pneumonia (PCP) and cytomegalovirus infection). Our case of PCP was induced by low-dose tacrolimus (0.5 mg/day) and low-dose methylprednisolone (mPSL; 4.0 mg/day) administered in a RA patient. We herein report the first case of PCP caused by low-dose tacrolimus.

## Case presentation

**Figure 1:**
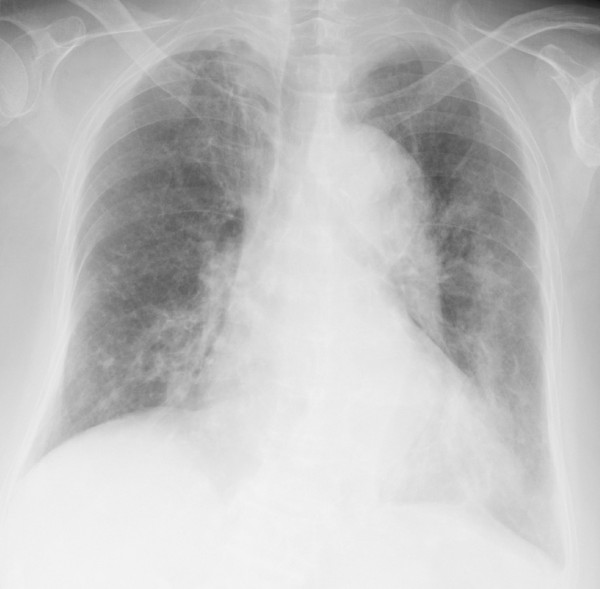
Chest X-ray obtained on admission showed consolidation in the left middle and lower lung fields.

**Figure 2:**
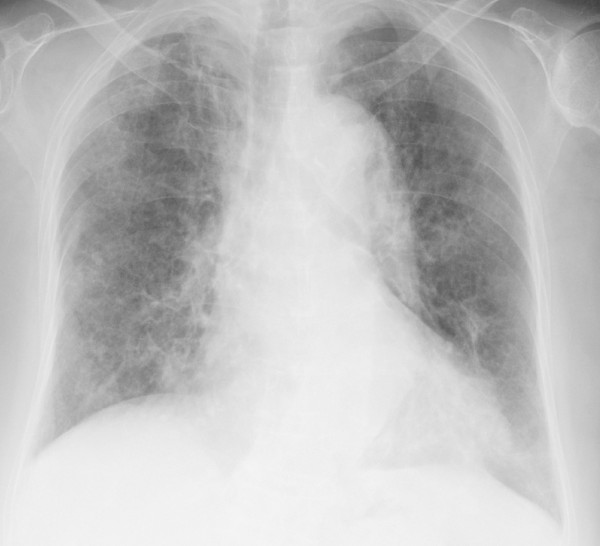
Chest X-ray obtained after the diagnosis of pneumocystis pneumonia showed areas of ground-glass opacity bilaterally in almost all lung fields.

**Figure 3:**
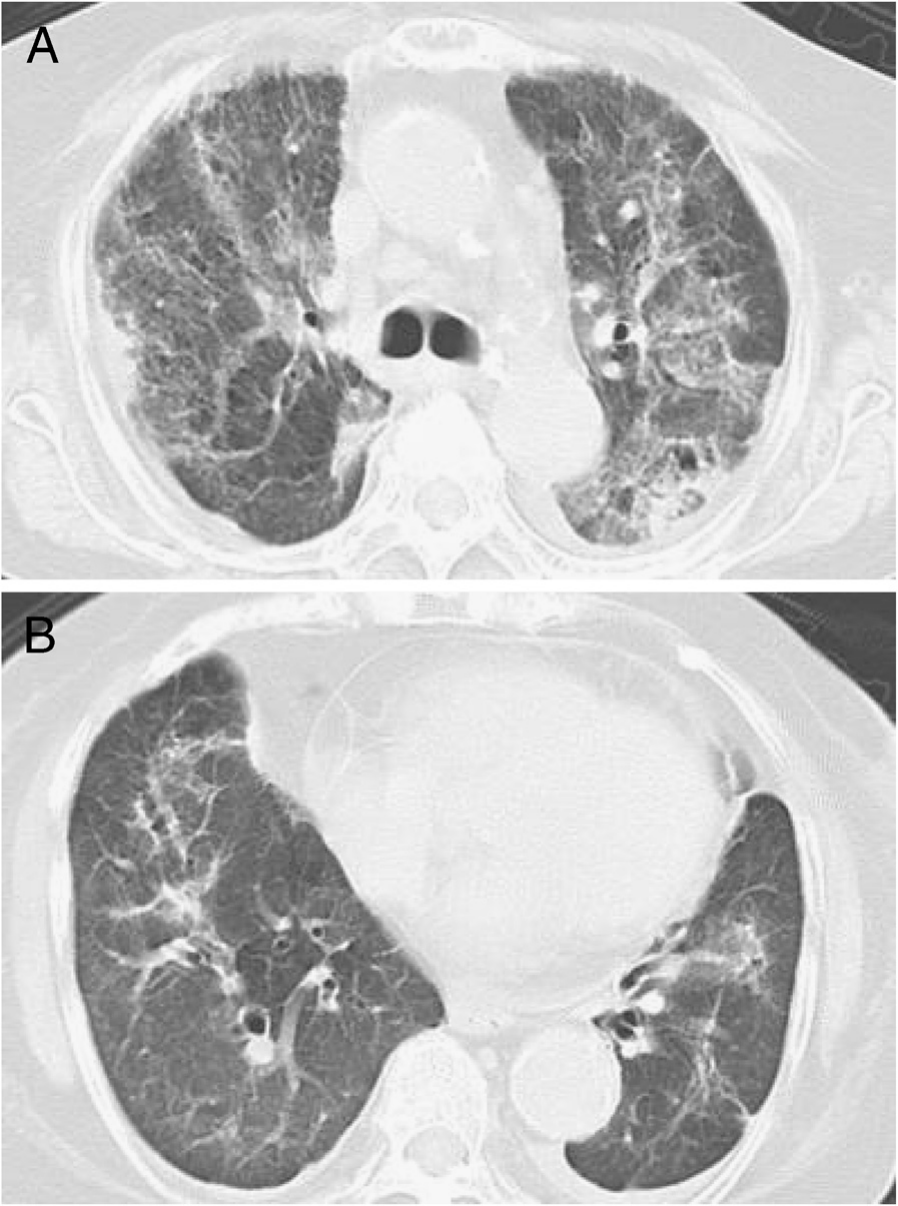
A chest computed tomography scan obtained after the diagnosis of pneumocystis pneumonia (upper lung field: 3A, lower lung field: 3B) showed areas of nonsegmental ground-glass opacity and subpleural curvilinear shadows bilaterally in all lobes.

An 82-year-old Asian Japanese female presented to our hospital with shortness of breath and was admitted for pneumonia. She had felt ill and had a cough for one week before admission. Her dyspnea had worsened and her cough had progressed with the development of hemosputum three days prior to presentation. Her past medical history was significant for RA and five prior admissions for pneumonia and bronchiectasis. She had been diagnosed with RA at 52 years of age and had been administered oral low-dose mPSL and salazosulfapyridine. Her general condition had been stable under this same treatment for 30 years; however, her arthralgia worsened three months before admission. The salazosulfapyridine was changed to low-dose tacrolimus (0.5 mg/day) by her physician, and her arthralgia almost completely disappeared. Her initial vital signs were as follows: body temperature, 38.0°C; blood pressure, 132/74 mmHg; heart rate 96 beats/minute; respiratory rate, 22 breaths/minute; and oxygen saturation (SpO_2_) on room air, 82%. A physical examination revealed stridor and bilateral basal expiratory wheezes. The laboratory test values were as follows: white blood cells, 8,400/μL with a left shift; lymphocytes, 740/μL; serum lactate dehydrogenase (LDH), 337 IU/L (normal, 130–220 IU/L); and serum C-reactive protein (CRP), 17.58 mg/dL (normal, < 0.3 mg/dL). A chest X-ray film (Figure 1) showed consolidation in the left middle lung field. A sputum Gram stain revealed many Gram-negative organisms, and the sputum culture grew *Pseudomonas aeruginosa*. Treatment with intravenous ceftazidime (2.0 g/day for nine days) was initiated, and the patient’s symptoms improved almost completely. On the ninth day of admission, the CRP level decreased from 17.58 to 4.00 mg/dL. On the 14th day of admission, the patient presented with severe dyspnea, and a physical examination was remarkable for an SpO_2_ of 82% on room air. The arterial blood gas values obtained on 5 L/min of oxygen delivered via nasal cannula were as follows: pH: 7.51, PaO_2_: 52 torr, PaCO_2_: 46 torr and bicarbonate: 27 mg/dL. A chest X-ray film (Figure 2) showed areas of ground-glass opacity (GGO) bilaterally in almost all lung fields. A chest computed tomography (CT) scan (Figure 3A,B) revealed areas of nonsegmental GGO and subpleural curvilinear shadows with predominance in the upper lobes in both lungs. The laboratory test values on the 14th day of admission were as follows: white blood cells, 5270/μL with a left shift; lymphocytes, 210/μL; serum LDH, 315 IU/L; serum CRP, 25.8 mg/dL; Krebs von den Lungen-6 (KL-6) level, 457 IU/L (normal, < 500 IU/L); surfactant protein-D (SP-D), 358 ng/mL (normal, < 110 ng/mL); plasma (1 → 3) beta-D-glucan level, 716 pg/dL (normal, < 11 pg/mL), which was high compared with the data obtained before this admission (17 pg/mL). Assays for latex-agglutination-Candida antigens and Aspergillus galactomannan antigens in the serum were both negative. The sputum, urine and blood cultures grew no organisms. The patient’s sputum was positive for *Pneumocystis jirovecii* according to polymerase chain reaction (PCR). She was immediately treated with sulfamethoxazole/trimethoprim (ST) at a dose of 10 mg/kg per day and high-dose mPSL (1 g/day for 3 days) with empirical antibiotic therapy (ciprofloxacin). However, her respiratory status rapidly deteriorated and she died on the 16th day of admission.

## Discussion

To our knowledge, this is the first case report of PCP induced by low-dose tacrolimus and steroid therapy for RA. Tacrolimus is an immunosuppressive drug that was discovered in Japan. Tacrolimus prevents the dephosphorylation of calcineurin and suppresses the production of inflammatory cytokines (i.e., interleukin (IL)-2, IL-3, IL-4, interferon-gamma and tumor necrosis factor (TNF)-alpha from T-cells). It is used to prevent acute rejection following organ transplantation and to treat autoimmune disease. In Japan, approximately 12,000 patients with RA have been treated with tacrolimus since 2005. The efficacy of tacrolimus has been demonstrated by many researchers, and the potential toxicity of this drug has also been reported. The major side effects of tacrolimus include renal dysfunction, interstitial pneumonia, dyspnea, infectious diseases, hyperglycemia, skin rashes and gastric dysfunction (e.g., nausea and abdominal pain). Regarding RA patients with interstitial pneumonitis, tacrolimus has been reported to be associated with either improvement or acute exacerbation of interstitial pneumonia. Ando, *et al*. reported a case of progressive interstitial pneumonia associated with amyopathic dermatomyositis refractory to cyclosporine that was successfully treated with tacrolimus [1]. Miwa, *et al*. reported a case of tacrolimus-induced lung injury in an RA patient with interstitial pneumonia [2]. In addition, Koike, *et al*. reported 27 cases of tacrolimus-induced lung injury [3]. However, to the best of our knowledge, even among these cases, there has so far been no report of lung injury caused by PCP related to low-dose tacrolimus therapy, while there have only been a few reports of PCP in RA patients under tacrolimus and low-dose mPSL therapy. With diseases other than RA, PCP during tacrolimus therapy has been reported in a patient after transplantation, and also two patients with UC [4]. In the setting of organ transplantation, the incidence of PCP is 4.8 cases per 1,000 person-transplant years [5,6]. No significant risk of PCP was reported in patients under tacrolimus therapy. However, combined treatment with tacrolimus and sirolimus carries a significantly higher risk of PCP than other types of combination therapy. Among the UC cases, one patient was medicated with high-dose tacrolimus (0.5 mg/kg/day) and mPSL, while the other was treated with tacrolimus (applied dose: not described) and 6-mercaptopurine. Both patients died from acute respiratory failure. Patients with human immunodeficiency virus (HIV) and autoimmune disease under the administration of high-dose steroids and immunosuppressants are likely to develop PCP. The administration of methotrexate or TNF-alpha inhibitors for autoimmune disease is associated with a higher risk of PCP than other treatments. In normal situations, the dose of tacrolimus is 3.0 mg/day; however, our patient was treated with low-dose tacrolimus and mPSL. To the best of our knowledge, there are no previous reports describing PCP induced by such a low dose of tacrolimus in the literature.

We diagnosed the patient with PCP due to the high level of beta-D-glucan and positive findings for *Pneumocystis jirovecii* in the sputum on PCR testing. Tasaka, *et al*. suggested that a diagnosis of PCP should be made based on a beta-D-glucan level above 31.1 mg/dL and positive findings for *Pneumocystis jirovecii* in bronchial alveolar lavage fluid (BALF) on PCR testing [7]. However, we were unable to perform bronchoscopy in our case due to the patient’s severe acute progression of respiratory failure. Therefore, we substituted findings of *Pneumocystis jirovecii* in the sputum on PCR testing for those in BALF. Tokuda, *et al*. reported that RA-related PCP exhibits both diffuse homogenous GGO with sharp demarcation in the interlobular septa and homogenous or inhomogeneous GGO with sharp demarcation in the intralobular septa on chest CT [8]. The chest CT findings observed in our case revealed non-homogenous GGO without interlobular septal boundaries in accordance with RA-related PCP. We administered steroid pulse therapy with ST because the rapid progression of respiratory failure did not allow us to sufficiently examine and differentiate the etiology of the disease in this case. Our patient was diagnosed with and treated for Pseudomonas pneumonia before diagnose of PCP was made. John, *et al*. reported that blood CD4+ lymphocyte counts in PCP patients with HIV were lower than 200/μL [9]. Enomoto, *et al*. reported that average lymphocyte counts in 17 RA patients complicated with PCP were 890/μL at the time of PCP diagnosis [10]. In our case, blood lymphocyte count was 1700/μL at the time of diagnosis of Pseudomonas pneumonia, however, that was 210/μL at the time of PCP diagnosis. The decreased lymphocyte count was considered to be due to the Pseudomonas pneumonia. Therefore, it was considered that some of the changes that occur in the immune response due to the effects of Pseudomonas pneumonia have the potential to trigger PCP. RA-related PCP has a higher mortality rate than HIV-related PCP [11]. It is assumed that providing diagnosis and treatment in the early stage of development of PCP achieves prognostic improvements in RA patients. Our patient rapidly developed respiratory failure following diagnosis and treatment.

## Conclusions

In conclusion, we herein reported the first case of PCP induced by low-dose tacrolimus and steroid treatment for RA. Clinicians should be careful to watch for the development of PCP following the administration of tacrolimus even in small doses in elderly patients and patients with a history of lung disease.

## Consent

Written informed consent was obtained from the patient’s next-of-kin for publication of this case report and any accompanying images. A copy of the written consent is available for review by the Editor-in-Chief of this journal.

## Abbreviations

RA: Rheumatoid arthritis; mPSL: Methylprednisolone; PCP: Pneumocystis pneumonia; SpO2: Oxygen saturation; LDH: Lactate dehydrogenase; CRP: C-reactive protein; CT: Computed tomography; GGO: Ground-glass opacity; KL-6: Krebs von den Lungen-6; SP-D: Surfactant protein-D; PCR: Polymerase chain reaction; ST: Sulfamethoxazole/trimethoprim; IL: Interleukin; TNF: Tumor necrosis factor; UC: Ulcerative colitis; HIV: Human immunodeficiency virus; BALF: Bronchial alveolar lavage fluid.

## Competing interests

The authors declare that they have no competing interests.

## Authors’ contributions

MK and KT reviewed the clinical data and were major contributors in writing the manuscript. YF, KS, HI, and KT were involved with patient management. HT performed the radiological examination of the chest x-ray and CT. All authors read and approved the final manuscript.
